# 3-D ultrastructure and collagen composition of healthy and overloaded human tendon: evidence of tenocyte and matrix buckling

**DOI:** 10.1111/joa.12164

**Published:** 2014-02-09

**Authors:** Jessica Pingel, Yinhui Lu, Tobias Starborg, Ulrich Fredberg, Henning Langberg, Anders Nedergaard, MaryAnn Weis, David Eyre, Michael Kjaer, Karl E Kadler

**Affiliations:** 1 Faculty of Health and Medical Sciences, Institute of Sports Medicine, Bispebjerg Hospital and Centre for Healthy Aging, University of CopenhagenCopenhagen, Denmark; 2 Wellcome Trust Centre for Cell-Matrix Research, University of ManchesterManchester, UK; 3 Diagnostic Centre, Region Hospital SilkeborgSilkeborg, Denmark; 4 Department of Orthopaedics & Sports Medicine, University of WashingtonSeattle, WA, USA

**Keywords:** 3View®, collagen, cross-links, fibers, fibrils, serial block face-scanning electron microscopy

## Abstract

Achilles tendinopathies display focal tissue thickening with pain and ultrasonography changes. Whilst complete rupture might be expected to induce changes in tissue organization and protein composition, little is known about the consequences of non-rupture-associated tendinopathies, especially with regards to changes in the content of collagen type I and III (the major collagens in tendon), and changes in tendon fibroblast (tenocyte) shape and organization of the extracellular matrix (ECM). To gain new insights, we took biopsies from the tendinopathic region and flanking healthy region of Achilles tendons of six individuals with clinically diagnosed tendinopathy who had no evidence of cholesterol, uric acid and amyloid accumulation. Biochemical analyses of collagen III/I ratio were performed on all six individuals, and electron microscope analysis using transmission electron microscopy and serial block face-scanning electron microscopy were made on two individuals. In the tendinopathic regions, compared with the flanking healthy tissue, we observed: (i) an increase in the ratio of collagen III : I proteins; (ii) buckling of the collagen fascicles in the ECM; (iii) buckling of tenocytes and their nuclei; and (iv) an increase in the ratio of small-diameter : large-diameter collagen fibrils. In summary, load-induced non-rupture tendinopathy in humans is associated with localized biochemical changes, a shift from large-to small-diameter fibrils, buckling of the tendon ECM, and buckling of the cells and their nuclei.

## Introduction

Tendons are vital for human locomotion because they transmit force from contracting skeletal muscle to bony structures. Tendon tissue is comprised of a relatively small population of fibroblast-like cells (tenocytes) surrounded by a collagenous extracellular matrix (ECM) that comprises ˜ 85% of the mass of mature tendon (reviewed by Kjaer, 2004). Tendon injuries represent a significant clinical problem, and details of the pathological changes in overloaded tendon and the associated development of tendinopathy are sparse (Arnoczky et al. 2007). Focal histopathological changes have been documented in the region of the overused tendon, including collagen disorganization, increased cellularity, cell rounding and neovascularization (de Mos et al. 2009). Further, it has been shown that expression of both collagen type I, collagen type III, matrix metalloproteinases and other ECM proteins is higher in tendinopathic than in healthy tendon (Jones et al. 2006; de Mos et al. 2007), and that the tissue content of several proteins like versican and aggrecan is upregulated in tendinopathy (Parkinson et al. 2010). Whether or not tendinopathy is associated with inflammatory activity is debated (Millar et al. 2009; Pingel et al. 2013a), and may depend upon the chronicity of the condition. However, the 3-dimensional (3D) changes in tendon structure and the collagen content of type I and III with tendinopathy are unknown.

Studies of the 3D organization of collagen fibrils *in vivo* are made experimentally difficult because of the narrowness of the fibrils (ranging from ˜ 12 to ˜ 500 nm). As a consequence, light microscopy has insufficient resolution for fibril diameter measurements and cannot distinguish individual fibrils within the crowded environment of a fibril bundle. The curvilinear organization of collagen fibrils in bundles, and the extreme lengths of collagen fibrils in comparison to the length of the cell, also present significant technical challenges to studies of organization; estimates of fibril length range from ˜ 1 μm to several millimeters (Craig & Parry, 1981). Transmission electron microscopy provides sufficient resolution to determine fibril diameter distributions, but serial section approaches are required to visualize long-range organization. However, difficulties in obtaining undistorted sections and in series have precluded a detailed ultrastructural study of tissue organization in tendinopathy. Recent application of serial block face-scanning electron microscopy (SBF-SEM) to studies of embryonic tendon tissue has shown that this technique is particularly useful for studying long-range collagen fibril organization (Starborg et al. 2013).

We hypothesized in this study that within the same human tendon a tendinopathic area of the tissue would demonstrate 3D structural disorganization of the tissue. We also predicted an increase in the ratio of collagen III : I. Type III collagen is a minor component of the predominately type I collagen-containing collagen fibrils that are widespread in human tissues (Fleischmajer et al. 1990a), and is synthesized in response to injury where it occurs as reticular (small diameter) collagen fibrils (Whitby & Ferguson, 1991). Here, we examined the collagen III and I content as well as the 3D ultrastructure of tendinopathic and non-tendinopathic regions of tendons using transmission electron microscopy and SBF-SEM.

## Materials and methods

### Participants

Six patients (age: 48 ± 6 years, mean and SE) suffering from chronic Achilles tendinopathy with focal mid-tendon pain, tendon thickening and ultrasonography-verified structural changes were recruited for this study. The recruitment of patients and isolation of biopsy material was approved by the regional ethical committee for The Capital Region of Copenhagen (H-1-2009-114). The subjects were either recreational athletes or manual workers with a history of Achilles tendon pain for more than 6 months (range 0.5–3 years), and they had all tried conventional treatments [i.e. eccentric strength training, oral anti-inflammatory drugs (non-steroidal anti-inflammatory drug, NSAID), peri-tendinous glucocorticoid injection] for tendinopathy without effect. Glucocorticoid injection or NSAID intake was not allowed for 6 months prior to the study. Subjects were recruited from the Department of Rheumatology, Silkeborg Hospital, Denmark.

### Tissue collection

Biopsies of Achilles tendon were obtained as a standard routine diagnostic procedure in order to detect deposits of cholesterol, uric acid and amyloid; none was detected in any of the individuals examined. The subjects had two tendon biopsies taken in the diseased Achilles tendon, one where the focal tendinopathy changes and symptoms were present, and one biopsy in a presumably healthy area of the same tendon. Excess material from the tendon biopsies was used for this study, with patient consent. There was sufficient material from all subjects for protein analysis and from two subjects for electron microscopy investigation. In brief, 2 × 2 × 2 mm biopsies were obtained under local anesthesia applied peri-tendinously from both the medial and lateral side of the tendon with ultrasound-guided injection of 10 mL 1% lidocaine on both sides. The biopsies were obtained under ultrasonographic guidance using a semi-automatic biopsy needle (14 GA, 9 cm; Angiotech). The initial biopsy was obtained in the tendon area with the most increased tendon thickness, hypoeccogenicity and potential neovascularization located typically 3–5 cm above the calcaneic insertion of the tendon. The second biopsy was obtained 4 cm proximal to the first biopsy in an ultrasonographic normal region of the tendon.

### Electron microscopy

Biopsy samples were prepared for electron microscopy as described previously (Starborg et al. 2013). In brief, 1 × 1 × 1 mm cubes of tendon were immersed in 2.5% glutaraldehyde prepared in 100 mm cacodylate buffer (pH 7.2), and processed using a double osmium protocol that is suitable for transmission electron microscopy and SBF-SEM (Starborg et al. 2013). Semi-thin (˜ 1 μm thick) sections were prepared, stained with toluidine blue and examined using a dissecting microscope to determine the orientation of the tendon within the biopsies. The resin blocks were trimmed for transverse sectioning (i.e. 90° to the tendon long axis). Ultrathin sections (70 nm thick) were prepared, and fibril diameter measurements were made using a FEI BioTwin transmission electron microscope. Resin blocks were trimmed and images were collected using an FEI Quanta 250 ESEM equipped with a Gatan 3View® for in-chamber ultramicrotome sectioning and image acquisition, as described previously (Starborg et al. 2013). Typically, 500–1000 × 100-nm-thick cuts were removed from the blocks during the imaging procedure, and image analysis and model reconstruction was performed using IMOD (Kremer et al. 1996).

### Protein analysis

Tissue samples for protein analysis were snap-frozen in liquid nitrogen and stored at −80 °C prior to analysis. Collagen was extracted by pepsin, and run based on equal dry weight loads of starting tissue on 6% sodium dodecyl sulfate–polyacrylamide gel electrophoresis using an interrupted electrophoresis method that resolves type III collagen chains (Wu et al. 2010, adapted to the method of Laemmli et al. 1970). Individual protein bands after Coomassie Blue staining were digested in-gel by trypsin (Kinter & Sherman, 2008; Eyre et al. 2008). The resulting peptides were subjected to microbore C8 column liquid chromatography (0.3 mm × 15 cm; Vydac) interfaced directly to a ThermoFinnigan LCQ Deca XP tandem mass spectrometer equipped with an electrospray ionization source. For protein identification, peptide fragments were compared with the NCBI non-redundant protein database using SEQUEST, an automated database search algorithm designed for use with tandem mass spectrometry (MS/MS) data.

## Results

### Elevated collagen III : I ratio extracted from tendinopathic tendon

**Figure 1:**
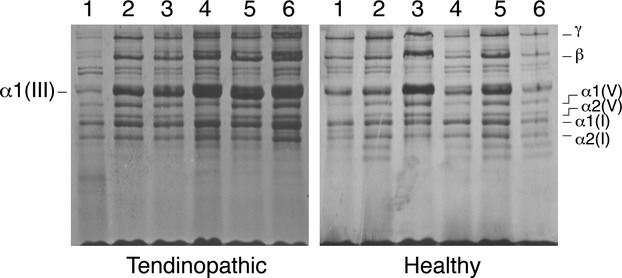
Sodium dodecyl sulfate–polyacrylamide gel electrophoresis of pepsin-solubilized collagens from healthy and tendinopathic sites in six individual patient tendons. The identities of the alpha chains of type I, III and V collagen are as indicated and were confirmed by mass spectroscopy.

As a first experiment we examined the ratio of collagen III : I extracted by pepsin from healthy and tendinopathic regions. Samples were examined at equal loads of starting tissue using an interrupted electrophoresis method that resolves type III collagen chains from types I and V collagen chains (Wu et al. 2010). As shown in Fig. 1, polypeptide chains from type I, III and V collagens were well resolved and migrated at expected relative locations. The identities of the proteins were confirmed by in-gel trypsin digestion and tandem mass spectrometry. The results showed that there was generally more type III collagen in extracts of samples of tendinopathic regions of tendon relative to type I collagen in the same extract, compared with the healthy regions that flanked the injury. Beta dimers and higher order cross-linked type III collagen bands were also evident, as were type V collagen chains. We note that type III collagen is probably fully extracted from the tissue, whereas the heavily cross-linked bulk type I collagen is only partially solubilized. Thus, the apparent ratio of collagen III : collagen I chains in the gels is probably higher than it is in tissue. Nevertheless, the results show a distinct difference between ‘healthy’ and ‘tendinopathic’ samples.

### Healthy and tendinopathic regions of tendon show marked differences in the distribution of fibril diameters: increased frequency of narrow-diameter collagen fibrils in tendinopathic regions

**Figure 2:**
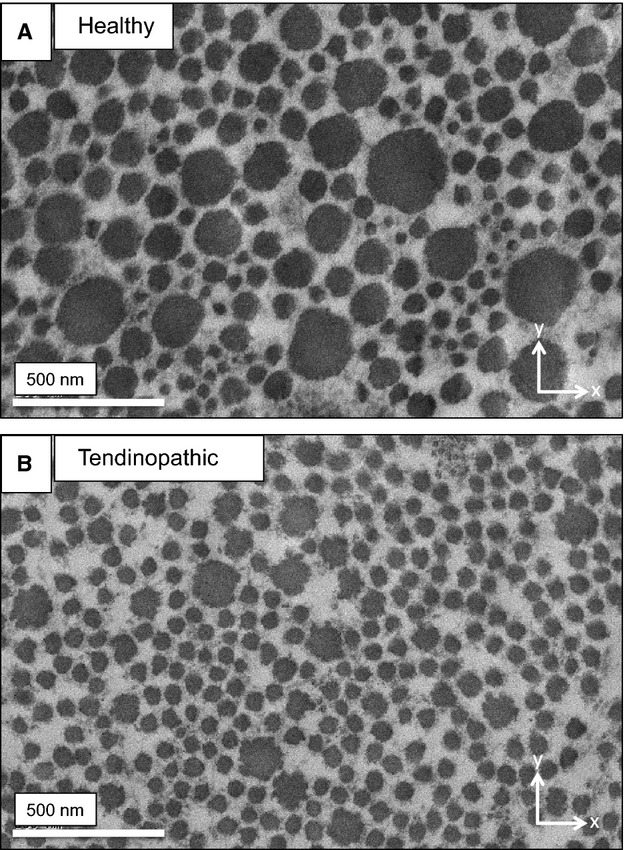
Transmission electron microscopy. Typical electron microscope images of normal (A) and tendinopathic (B) tendon. Arrows indicate the axes of orientation, with *x* and *y* representing a plane at right angles (transverse) to the long axis of the tendon.

**Figure 3:**
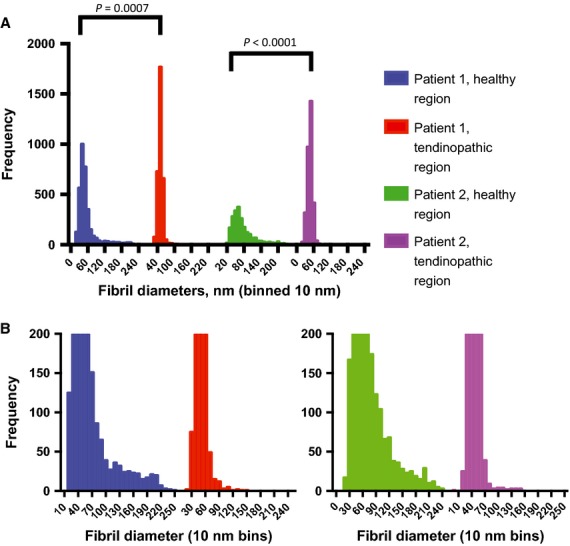
Fibril diameter distributions in healthy and tendinopathic regions. (A) The frequency distributions of collagen fibril diameters from the healthy regions and tendinopathic regions of tendon from two patients are shown. (B) The data are re-plotted to illustrate the distribution of large-diameter fibrils, which are most evident in healthy regions. *P*-values show significant differences between healthy and tendinopathic regions (Mann–Whitney *U* non-parametric tests).

As a next experiment we quantified collagen fibril diameters in healthy and tendinopathic regions of two different individuals using transmission electron microscopy. The tendon biopsies were embedded for electron microscopy, and the resin blocks oriented with the long axis of the tendon parallel to the long axis of the trimmed pyramid. Typical transmission electron microscopy images are shown in Fig. 2, and the analysis of fibril diameters is show in Fig. 3. The results showed an increased number of small-diameter and decreased number of large-diameter fibrils in tendinopathic regions compared with healthy tendon. The minimum diameter of fibrils was in the range ˜ 17–23 nm in healthy and tendinopathic regions. However, the most noticeable difference was in maximum diameter, which was ˜ 241–246 nm in healthy regions and ˜ 150–154 in tendinopathic regions. The results are summarized in Table 1.

**Table 1:** Fibril diameter data.

	Patient 1 healthy region	Patient 1 tendinopathic region	Patient 2 healthy region	Patient 2 tendinopathic region
Total number of values	3430	3319	2215	3229
Minimum	17.03	23.461	18.404	17.761
Median	45.234	49.769	63.171	46.855
Maximum	246.037	154.819	241.745	150.267
Mean	55.269	50.1685	74.8622	46.7616
SD	34.9668	8.72429	40.5007	10.6447
SEM	0.597048	0.151435	0.860548	0.187326

### Marked differences in the organization of the ECM and the shape of the cells in healthy and tendinopathic regions

**Figure 4:**
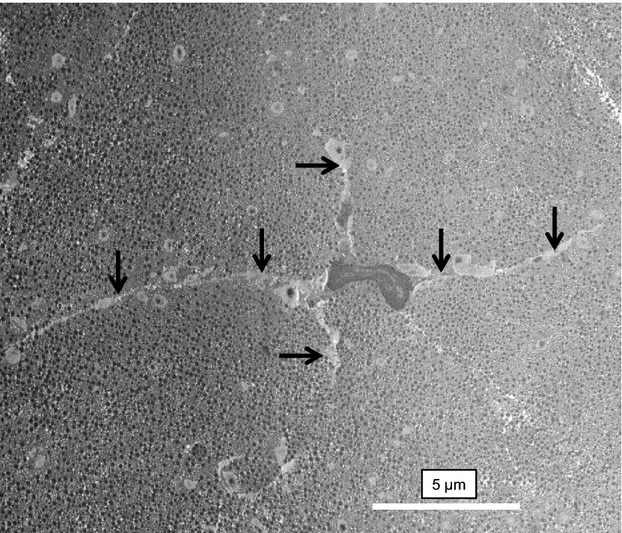
Tenocytes in healthy tendon. SBF-SEM image of a tenocyte in a healthy region of tendon. The cell is surrounded by a well-organized ECM containing collagen fibrils. Arrows indicate long cellular processes that project deep into the ECM.

**Figure 5:**
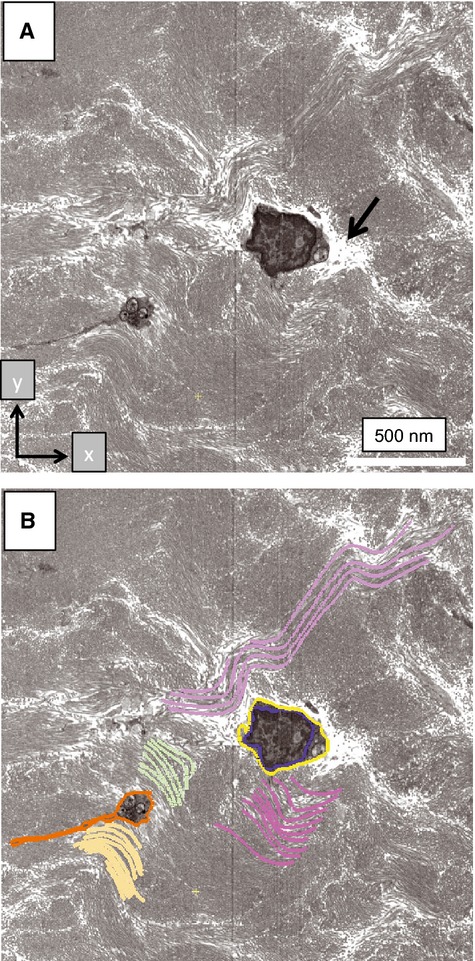
Disorganization of the ECM in tendinopathic regions. Tendinopathic tissue was examined by SBF-SEM and subsequently analyzed by IMOD. (A) Section 450 (of 740 × 100-nm-thick sections) showing cross-sections of two cells that are surrounded by curvilinear bundles of collagen fibrils. Arrow indicates electron-lucent regions between cells and the surrounding ECM. (B) Segmentation of some of the objects shown in (A). Blue, nuclear membrane. Yellow and orange, plasma membranes. Purple, green and yellow, collagen fibril bundles. Two cells are segmented (one colored orange, the other colored yellow). The nucleus of the cell bounded in orange is not visible because it is located at a different *z*-height in relation to the cell bounded in yellow.

**Figure 6:**
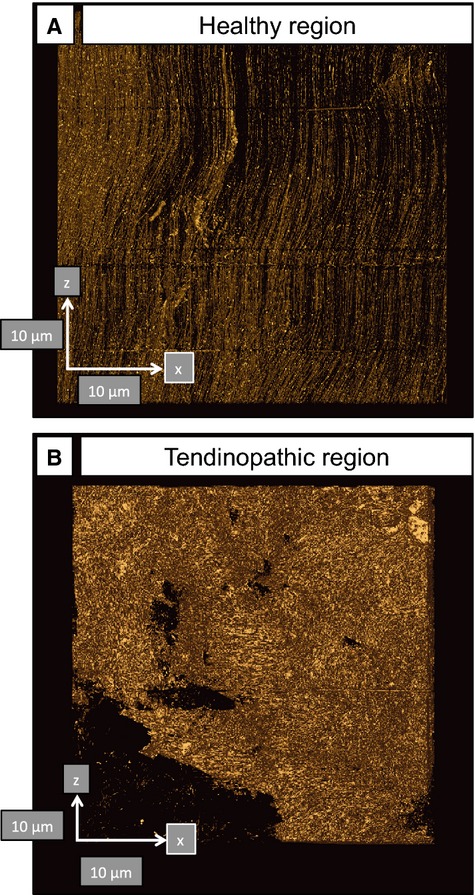
Automated segmentation in IMOD identifies changes in the organization in normal and tendinopathic regions of the same tendon. (A) Healthy tendon shows near-parallel alignment of collagen fibrils (oriented north–south). (B) Tendinopathic tendon contains disorganized ECM. Arrows show orientation and are 10-μm scale bars.

Using previously described methods (Starborg et al. 2013), we analyzed the healthy and tendinopathic regions by SBF-SEM, which was used to image the block face and to make 100-nm-thick cuts that were transverse (i.e. 90°) to the tendon long axis. The digital images of the block face were stacked and analyzed in IMOD (Kremer et al. 1996) to make 3D reconstructions. In the healthy tendon regions the tenocytes exhibited long processes that projected deep into the ECM that contained well-organized collagen fibrils (Fig. 4; Video S1). In tendinopathic regions the long processes were absent and the cells were separated from the ECM by electron-lucent regions (Fig. 5a), not seen in healthy tendon. Electron-lucent regions also occurred between bundles of collagen fibrils that were often disorganized (Video S2). Close examination of the electron-lucent regions of tendinopathic tendon showed local distortions in the fibril bundles. Collagen fibrils traversed across the tendon long axis and exhibited local high curvature. In further analysis we used segmentation tools within IMOD to identify collagen bundles in healthy and tendinopathic tendon (Fig. 5b). Using automated segmentation tools in IMOD, we performed an objective analysis of the organization of the ECM in healthy and tendinopathic regions. As shown in Fig. 6(a, healthy; b, tendinopathic), the linear organization of the tendon ECM was severely disrupted in the tendinopathic region.

### Cell sliding and buckling in tendinopathic tendon

**Figure 7:**
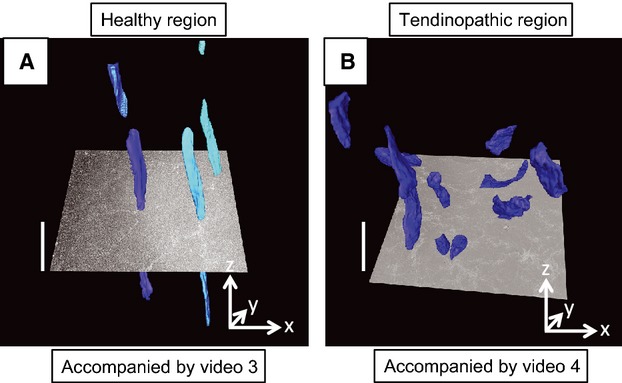
Three-dimensional organization of tenocytes in healthy and tendinopathic regions. (A) Healthy tendon. Nuclei are shown in different hues of blue and are aligned parallel to the tendon long axis. (B) Tendinopathic region of tendon. Nuclei are distributed almost randomly in three-dimensions. The images are frame shots of Videos S3 and S4. An electron microscope image is shown superimposed on the 3D reconstructions. The sizes of the images are 40 × 40 μm (*x*–*y*-axes). The scale bars on the *z*-axis (i.e. parallel to the tendon long axis) are 10 μm.

**Figure 8:**
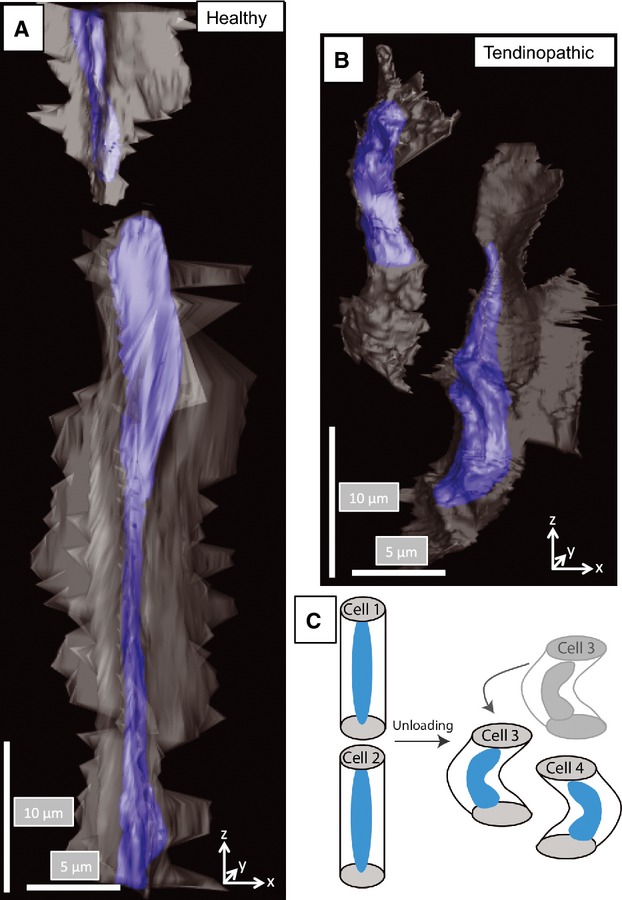
Buckling and slippage of cells in tendinopathic regions. (A) Three-dimensional reconstruction showing two cells in healthy tendon. The large cell in the center of the view is undergoing mitosis and is aligned parallel to the tendon long axis. Both cells are stacked one-on-top of the other. (B) Three-dimensional reconstruction showing two cells in tendinopathic tendon. The cells are aligned side-by-side, which was not observed in healthy tendon. (C) Schematic representation of cell slippage to explain the change in cell orientation from head-to-tail to side-by-side alignment.

Next, we used SBF-SEM image stacks representing 40 × 40 × 750 μm (*x*–*y*–*z*-axes, with the *z*-axis parallel to the tendon long axis) volumes of tendon and IMOD for image analysis, to examine the cellular alignment in healthy and tendinopathic regions. Typical 3D reconstructions are shown in Figs 6 and 7. In healthy tendon the cells were aligned in stacks along the tendon long axis (Fig. 7a; Video S3). However, cells in tendinopathic regions were positioned mostly beside each other (Fig. 7b; Video S4). Examination of the 3D reconstructions from side-on showed that the cells in the tendinopathic regions had misshapen nuclei compared with cells in healthy tendon (Fig. 8a,b). The misalignment of the cells, the distorted nuclei and the disorganization of the collagen bundles suggested slippage of the cells past one another, as shown schematically in Fig. 8c.

## Discussion

The two major observations in the present study are: (i) the parallel alignment of the ECM that is seen in healthy tendon is markedly perturbed in tendinopathic regions of the same tendon; and (ii) tenocytes undergo shortening and buckling in tendinopathic regions. Here, instead of the cells being stacked in rows as has been observed previously (Ralphs et al. 1998), they occur side-by-side and contorted. A further and unexpected observation was the misshapen cell nuclei in tendinopathic regions (e.g. see Fig. 8). The simplest explanation for the contorted nuclei, misaligned cell bodies and misaligned collagen fibril bundles was buckling of the ECM in the absence of tensional load. The reason for buckling is not fully understood, but is most likely the result of local breakage of collagen fibrils with concomitant contraction of the actinomyosin cytoskeleton. Recent studies using a tendon-like construct system showed that cells exert forces on the surrounding ECM (Herchenhan et al. 2012; Kalson et al. 2013). In the absence of sufficient restraining force in the matrix (as might occur if a small but significant number of fibrils are severed), cell contraction can result in matrix buckling and nucleus shape changes.

We found that the distribution of fibril diameters in the tendinopathic part of the tendon exhibited a shift to smaller diameters, which was due to a lack of large-diameter fibrils and the increased occurrence of small-diameter fibrils in tendinopathic regions. This observation contrasts with findings from insertional patella tendinopathy where more large-diameter fibrils were found (Kongsgaard et al. 2010). Both the fact that different tendons were used and that tendon biopsies in the patella could not always be obtained in the de-facto insertional part where the pathological ultrasonographic signal is found and where the clinical symptoms are located, could explain differences in findings of fibril diameter between studies. Further, the fact that subjects in the latter study have had tendinopathy for a relatively short period of time, vs. the individuals in the present study who had tendinopathy for years, can further contribute to the pronounced differences seen between studies. In the present study we cannot confirm that the shift to smaller diameters represents the formation of new fibrils or an altered turnover of existing fibrils. In the study by Pingel and co-workers (Pingel et al. 2012) on similar individuals as in the present study, no increased mRNA for scleraxis or tenomodulin was found, which to some extent indicates a lack of new fibril formation. On the other hand, in that study both expression of collagen type I and III as well as of matrix metalloproteinases were upregulated, which could indicate an increased tissue turnover in tendinopathic tendon compared with the healthy part.

The occurrence of small-diameter fibrils and the increased abundance of type III collagen (after pepsinization) in tendinopathic tissue could potentially be explained by an increase in type III pNcollagen. (Note that pNcollagen molecules in the tissue would have been converted to collagen by the pepsin treatment used to extract the proteins from the tissue.) The observation of increased type III collagen fits with the finding of higher type III collagen content in tendinopathy vs. normal healthy tendon. It has been shown that type III pNcollagen is the main tissue form that can polymerize on the surface of type II collagen fibrils in articular cartilage in osteoarthritis (Wu et al. 2010) and on type I collagen in skin (Fleischmajer et al. 1990b). Thus, it is likely that in tendinopathy, type III collagen formation dominates and attaches to type I collagen fibrils in the structural adaptation to tendinopathy, most likely as type III pNcollagen.

Although we can be confident that the cells in healthy tendon were tenocytes, we cannot be certain about the type of cells in tendinopathic regions or their origins. For example, cells could have invaded the tendinopathic area from nearby tissues or be delivered by the circulation. No sign of inflammation was found in this long-term tendinopathy, which would fit with the view that inflammatory reactions occur only during the early stages of tendinopathy (Millar et al. 2010). Inflammatory cells have never been found in chronic tendinopathies, not even after acute exercise (Pingel et al. 2013b), in spite of the ultrasound-guided peritendinous corticosteroid injection that can normalize the ultrasound image of the thickened tendon, removing pain and neovascularization (Fredberg et al. 2004).

It has been proposed that in tendinopathy regions of disease, cells are ‘shielded’ from tension and are thereby subjected to de-tensioning (Arnoczky et al. 2007). However, the apparent upregulation of collagen type III relative to type I collagen observed on the protein level in this study is unlikely to be fully explained by just de-tensioning. In support of this, unloading presumably occurs along the entire length of the tendon, and not just at the site of clinical tendinopathy. Also, a recent study showed that in a tendon cell construct model that results in artificial tendon formation (Kapacee et al. 2008; Bayer et al. 2010), de-tensioning of the constructs was accompanied by downregulation of collagen gene expression, but did so in both collagen type III and I, and with no apparent change in the ratio between the collagen type I and type III expression (Bayer, under submission). Another possible reason for the upregulation of type III collagen was the dramatic change in cell shape, especially the nucleus, in tendinopathic regions. The role of cell shape and nucleus shape in regulation of gene expression is poorly understood but warrants further study.

## Conclusions

The present study demonstrated that overused tendon that develops tendinopathy in humans is associated with focal biochemical and structural changes of the tendon tissue. A model (Fig. 8c) is proposed in which local unloading in the tendon as a result of tendinopathy results in buckling of the matrix and cells. We propose that tension produced by tenocytes on the surrounding ECM is normally counterbalanced by tissue tension in the ECM. Breakage of a critical number of collagen fibrils removes sufficient tissue tension to allow cell-induced buckling of the tissue. The buckling of the tissue (matrix and cells) results in loss of the normal parallel alignment of the ECM and loss of the normal stacking of cells.

## Supporting Information

Video S1 Step-through movie generated from 550 images of SBF-SEM analysis of healthy tendon.

Video S2 Step-through movie generated from 550 images of SBF-SEM analysis of healthy tendon.

Video S3 Three-dimensional reconstruction of healthy tendon showing the nuclei of adjacent cells in head-to-tail alignment.

Video S4 Three-dimensional reconstruction of tendinopathic tendon showing the nuclei of adjacent cells. Disorganized cells are a feature of tendinopathic tendon.
