# Supplementary material for: 3-D ultrastructure and collagen composition of healthy and overloaded human tendon: evidence of tenocyte and matrix buckling
Source: J Anat. 2014 Feb 9;224(5):548–55. doi: 10.1111/joa.12164 (PMC3981497; doi:10.1111/joa.12164)
Supplement: Supplementary file 5 [file joa0224-0548-sd5.docx]

**Supplementary Data online**

**Video 1**

Step-through movie generated from 550 images of SBF-SEM analysis of healthy tendon.


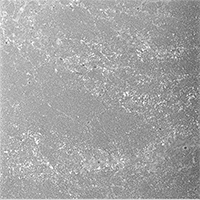


**Video 2**

Step-through movie generated from 550 images of SBF-SEM analysis of healthy tendon.


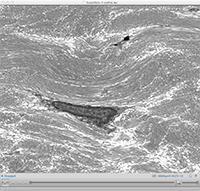


**Video 3**

3-Dimensional reconstruction of healthy tendon showing the nuclei of adjacent cells in head-to-tail alignment.


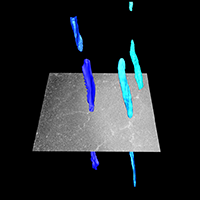


**Video 4**


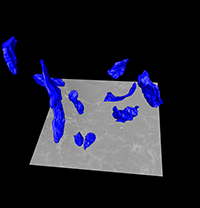
3-Dimensional reconstruction of tendinopathic tendon showing the nuclei of adjacent cells. Disorganized cells is a feature of tendinopathic tendon.
